# Promoter DNA Methylation of Farnesoid X Receptor and Pregnane X Receptor Modulates the Intrahepatic Cholestasis of Pregnancy Phenotype

**DOI:** 10.1371/journal.pone.0087697

**Published:** 2014-01-31

**Authors:** Romina Cabrerizo, Gustavo O. Castaño, Adriana L. Burgueño, Tomas Fernández Gianotti, María Mora Gonzalez Lopez Ledesma, Diego Flichman, Carlos J. Pirola, Silvia Sookoian

**Affiliations:** 1 Department of Clinical and Molecular Hepatology, Institute of Medical Research A Lanari-IDIM, University of Buenos Aires-National Council of Scientific and Technological Research (CONICET), Ciudad Autónoma de Buenos Aires, Argentina; 2 Liver Unit, Medicine and Surgery Department, Hospital Abel Zubizarreta, Ciudad Autónoma de Buenos Aires, Argentina; 3 Research Council in Health, Ciudad Autónoma de Buenos Aires, Argentina; 4 Department of Molecular Genetics and Biology of the Complex Diseases, Institute of Medical Research A Lanari-IDIM, University of Buenos Aires-National Council of Scientific and Technological Research (CONICET), Ciudad Autónoma de Buenos Aires, Argentina; 5 Department of Virology, School of Pharmacy and Biochemistry, University of Buenos Aires, Ciudad Autónoma de Buenos Aires, Argentina; Nihon University School of Medicine, Japan

## Abstract

The intrahepatic cholestasis of pregnancy (ICP) is a multifactorial liver disorder which pathogenesis involves the interplay among abnormal bile acid (BA) levels, sex hormones, environmental factors, and genetic susceptibility. The dynamic nature of ICP that usually resolves soon after delivery suggests the possibility that its pathobiology is under epigenetic modulation. We explored the status of white blood peripheral cells-DNA methylation of CpG-enriched sites at the promoter of targeted genes (*FXR*/*NR1H4*, *PXR*/*NR1I2*, *NR1I3*, *ESR1*, and ABCC2) in a sample of 88 ICP patients and 173 healthy pregnant women in the third trimester of their pregnancies. CpG dinucleotides at the gene promoter of nuclear receptors subfamily 1 members and *ABCC2* transporter were highly methylated during healthy pregnancy. We observed significant differences at the distal (−1890) and proximal promoter (−358) CpG sites of the *FXR*/*NR1H4* and at the distal *PXR/NR1I2* (−1224) promoter, which were consistently less methylated in ICP cases when compared with controls. In addition, we observed that methylation at *FXR*/*NR1H4*-1890 and *PXR/NR1I2-1224* promoter sites was highly and positively correlated with BA profiling, particularly, conjugated BAs. Conversely, methylation level at the proximal *FXR*/*NR1H4*-358 CpG site was significantly and negatively correlated with the primary cholic and secondary deoxycholic acid. In vitro exploration showed that epiallopregnanolone sulfate, a reported FXR inhibitor, regulates the transcriptional activity of *FXR*/*NR1H4* but seems to be not involved in the methylation changes. In conclusion, the identification of epigenetic marks in target genes provides a basis for the understanding of adverse liver-related pregnancy outcomes, including ICP.

## Introduction

Intrahepatic cholestasis of pregnancy (ICP) is a liver disorder characterized by the presence of pruritus and biochemical cholestasis, appearing predominantly during the third trimester of pregnancy and disappearing spontaneously after parturition. Although ICP poses little maternal risk, fetal complications, such as high frequency of abnormal intrapartum fetal heart rate and amniotic fluid meconium, can lead to prematurity, fetal distress, and intrauterine death [1], [2].

ICP is a complex phenotype, and its pathogenesis involves the interplay of a cluster of intricate risk factors, such as abnormal bile acid (BA) levels, an impaired sex hormone profile, and genetic susceptibility [3]–[6].

Likewise, environmental factors, such as uneven geographical distribution [4], [7], and dietary factors including seasonal fluctuations of mineral dietary components [8] and gut-derived endotoxins due to increased gastrointestinal permeability [9] were also associated with ICP.

This complex scenario prompted us to speculate that a close gene–environment interaction is a critical modifier of the ICP pathogenesis, and this interaction might be robustly modulated by epigenetic mechanisms. In addition, the dynamic nature of ICP that usually resolves soon after delivery reinforces that the biological plausibility about its pathobiology is under epigenetic modulation. Nevertheless, to our knowledge, no studies have provided data about whether epigenetic mechanisms are involved in the ICP phenotype.

Based on this assumption, we performed a hypothesis-driven exploration of the status of DNA methylation at the carbon-5 position of cytosine in CpG dinucleotides in the promoters of candidate target genes in a case-control study of ICP.

Target gene selection was based on the knowledge of genes that were associated with either the pathogenesis of ICP or involved in bile acid metabolism, such as farnesoid X receptor (*FXR/NR1H4*), pregnane X receptor (*PXR/NR1I2*, also known as steroid and xenobiotic receptor *SXR*, or pregnane-activated receptor *PAR*), and the constitutive activator of retinoid response CAR (nuclear receptor subfamily 1, group I, member 3 (*NR1I3)*. We also included a nuclear hormone receptor, estrogen receptor 1 (*ESR1*, also known as estradiol receptor, ER-alpha or nuclear receptor subfamily 3 group A member 1), and ATP-binding cassette (ABC), subfamily C (CFTR/MRP), member 2 (*ABCC2*), whose protein is expressed in the canalicular part of the hepatocyte, playing an important role in bile formation and detoxification; a common variant in exon 28 of *ABCC2* gene (rs3740066) was associated with genetic susceptibility of ICP in South American patients [10].

## Patients and Methods

### Ethics Statements

This study was performed according to the principles of the Declaration of Helsinki and was approved (Protocol and approval number 2124MSGC/2011) by the institutional review board and the bioethical committee of our institution (“Comite de Bioetica del Hospital Abel Zubizarreta, GCBA”). Furthermore, written consent was obtained from all the patients.

### Patients and Control Pregnant Women

We collected clinical data and blood samples from 88 ICP patients and 173 unrelated healthy pregnant women in their third trimester of pregnancy in a cross-sectional, case-control hospital-based study; during the same study period, for each ICP patient in the liver unit, two healthy pregnant women were matched and included in the analysis. The genetic background of both patients and controls was the same, and the control population was matched on ethnicity, area of residence, and time of recruitment with affected individuals [10].

Diagnosis of ICP was based on the following criteria: 1. presence of pruritus occurring during the second half of an otherwise uneventful pregnancy; 2. the presence of abnormalities in liver function test suggestive of ICP: serum levels of alanine aminotransferase (ALT) and aspartate aminotransferase (AST) >40 and >35 IU/L, respectively; 3. elevated levels of fasting total bile acid (BA) >12 µmol/L; 4. no skin lesions caused by systemic diseases that could cause pruritus; and 5. spontaneous resolution of clinical symptoms and laboratory findings after delivery. All the patients were referred during pregnancy.

Additional inclusion criteria were the absence of infection by hepatitis viruses (HAV, HBV, and HCV; AxSYM, Abbott Park, IL, USA), autoimmune diseases, moderate-to-severe alcohol intake, HIV infection (enzyme-linked immunoabsorbent assay; Organon Teknika), biliary obstruction, and the use of drugs or alternative medicine therapy known to precipitate cholestasis.

Maternity data including age, geographical origin, pregestational BMI, education, smoking (number of cigarettes/day), history of abortion, history of preterm birth and previous abnormal fetal weight, preeclampsia-eclampsia, hypertension in pregnancy, hyperlipidemia or diabetes, pregnancy weight gain, and weight of the previous offspring were evaluated. Besides, family history of the disease was also recorded.

Resting systolic and diastolic arterial blood pressure (SABP and DABP, respectively) was measured in all the mothers after they had been sitting for at least 30 min. A mercury sphygmomanometer was used to measure blood pressure three times at the right arm.

Homeostasis model assessment (HOMA-IR) was used to evaluate the insulin resistance index and was calculated as follows: Fasting serum insulin (µU/ml)×Fasting plasma glucose (mmol/l)/22.5.

None of the mothers had either preeclampsia-eclampsia or gestational diabetes, and none of them were taking any medication.

UDCA was prescribed at doses of 900 mg/day for all the patients with gestational age of <37 weeks until delivery; serum samples were taken at basal conditions at the time of diagnoses.

### Neonatal Characteristics

Complete medical, obstetrical, and perinatal data were recorded. Apgar score [11] and gestational age, assessed using the Capurro method, were registered in all newborns [12]. In addition, all relevant clinical variables, such as gender, cephalic perimeter, birth weight of the newborn, mode of delivery, and perinatal morbidity, were registered for each pregnancy. Signs of fetal distress, such as meconium-stained amniotic fluid or clinical or Doppler ultrasonographic signs of hypoxemia, low birth weight, spontaneous preterm delivery, Apgar score <7, small-for-gestational age, asphyxial events, operative delivery due to asphyxia, or Apgar score <7 at 5 min, were assessed as fetal complications. Small-for-gestational age was defined as the birth weight for gestational age less than the specific 10^th^ percentile cutoff of a published Argentinian fetal growth reference [13].

### Biochemical Determinations

In every case, serum samples were extracted after a fasting period of 8 h, and aliquots were frozen at −20^o^C until chromatographic studies were performed. ALT (normal value <31 IU/L), AST (normal value <46 IU/L), alkaline phosphatase (normal value <240 IU/L), γ-glutamyl transpeptidase (γ-GT) (normal value <32 IU/L) activities, and total and conjugated bilirubin concentrations were measured using routine automated techniques. Total BA, cholic acid (CA), deoxycholic acid (DCA), CDCA, and UDCA in their free, glycol, and tauro derivative forms were assessed using capillary electrophoresis in a subsample of 40 cases and 38 controls. The details of the analytical method have been published elsewhere [3].

### Molecular Methods: Analysis of DNA Methylation using Quantitative HpaII PCR Assay

Genomic DNA was extracted from the white blood peripheral cells (WBC) from a blood sample using a standard method as previously described [14].

After digestion of genomic DNA with the methylation-sensitive endonuclease HpaII, the methylation status of candidate gene promoters was assessed using a real-time quantitative PCR assay as described elsewhere [15], [16]. Leukocyte *Hpa*II-digested DNA and undigested DNA were PCR amplified with primers specific to each selected region of the candidate gene promoters; the primer sequences are listed in **Table S1.** An iCycler thermocycler (BioRad Hercules, CA, USA) was used for DNA methylation quantification, using SYBR Green (Invitrogen, Buenos Aires, Argentina) as a fluorescent dye. The results are expressed as the ratio of digested to undigested product in triplicate.

Putative methylation target at the 5′-cytosine residues of the dinucleotide CpG in the gene promoter of *FXR*/*NR1H4* (−358 and −1890), *PXR* / *NR1I2* (−471 and −1224), *NR1I3* (−2377), *ESR1* (−173), and *ABCC2* (−2438) located at positions relative to transcription starting site were explored. A sequence starting 2,500 bp upstream from the transcriptional start site (TSS) of candidate genes was used to search for regions with potentially methylated CpG sites. Each sequence was retrieved from the database of transcriptional start site at http://dbtss.hgc.jp/, with the following ID numbers: *FXR*/*NR1H4* (NM_005123), *PXR*/*NR1I2* (NM_003889.3), *NR1I3* (NM_001077469.2), *ESR1* (NM_001122742.1), and *ABCC2* (NM_000392.3).

### Cell Culture and Treatment Experiment

Huh7 human hepatoma cells were cultured in Dulbecco modified Eagle medium, supplemented with 10% (v/v) of fetal bovine serum (GIBCO) at 37°C and 5% CO_2_. The cells seeded in 6-well plates at 50% confluence were treated with the following drugs: 5 µM 5-Aza-2′-deoxicitidine (Sigma-Aldrich Co. LLC, Saint Luis, MO, USA), an agent that inhibits DNA methylation, and 100 µM 5α-Pregnan-3β-OL-20-one sulfate, sodium salt, also known as epiallopregnanolone sulfate PM5S (Catalog ID P3865-000, Steraloids, Newport, RI, USA). The culture supernatant was harvested 3 days after treatment and centrifuged at 3,000×g for 10 min at 4°C and stored at −70°C. The cells were washed twice with ice-cold phosphate-buffered saline and lysed on ice with lysis buffer (10 mM Tris-HCl, pH: 8.0; 0.05 M NaCl; 0.5% NP40; and 1 mM EDTA). From these supernatants, RNA and DNA were extracted immediately using trizol methods. Cell viability was evaluated using the MTS/PMS colorimetric method. The cells were incubated with MTS/PMS in the shelter of the light at 37°C. After an hour of incubation, the absorbance was measured at 490 nm.

### Statistical Analysis

For statistical analyses, STATISTICA program was applied with *P*<0.05 regarded as statistically significant. Normally distributed data were tested using Pearson correlations, Student's *t*-tests, or analysis of variance, depending on homogeneity of the variance. For non-normally distributed data, Spearman correlations, nonparametric Mann-Whitney U, and Kruskal-Wallis H tests were used. In particular, Wilcoxon matched paired test was used to compare variables, such as the methylation levels in two positions of the same promoter. Normality of distribution was assessed using Kolmogorov-Smirnov test. All tests were two-tailed. Categorical data were tested using chi-square test.

## Results

The clinical, biochemical, obstetrical, and perinatal characteristics of ICP patients and healthy pregnant women are shown in **Table 1**. As expected, ICP patients showed significant differences not only in BA but also in serum bilirubin levels and liver enzymes. Severe pruritus was observed in 79% of the patients, and no multiple pregnancies were observed in this group. Furthermore, 10% of the patients had previous episodes of obstetric cholestasis, and 16% of the patients had familial history of ICP. Labor at term was induced in 33% of the patients because of ICP, and Cesarean section was performed in 31% of the patients. **Table 2** shows the detailed BA profiling in patients with ICP and control pregnant women.

**Table 1:** Clinical, biochemical, obstetrical, and perinatal characteristics of patients with intrahepatic cholestasis of pregnancy (ICP) and healthy pregnant women.

Characteristics	ICP patients	Healthy pregnant controls	P level
Age (years)	26.0±6.1	27.6±6.6	NS
Number of pregnancies	2.57±1.92	2.58±1.84	NS
Gestational age at delivery (weeks)	36.27±4.64	34.62±4.65	NS
Neonate birth weight (gr.)	3395.1±543.4	2986.4±444.8	0.09
Neonate Apgar score	9.57±0.81	8.55±1.24	0.001
Total bilirubin (µmol/L)	0.41±0.22	0.75±0.38	<1×10^−8^
Direct bilirubin (µmol/L)	0.08±0.05	0.29±0.25	<1×10^−8^
Asparte aminotransferase (IU/L) AST	18.55±7.28	75.84±77.7	<1×10^−8^
Alanine amino transferase (IU/L) ALT	15.18±7.6	98.7±118.1	<1×10^−8^
Alkaline phophatase (IU/L)	251.3±121.9	651.9±283.1	<1×10^−8^
Gamma glutamil-transpeptidase (IU-L)	24.2±16.0	77.8±119.2	<1×10^−8^

Results are expressed as mean ± SD. P-level indicates statistical significance using Mann-Whitney test.

**Table 2:** Bile acid profile in healthy pregnant women and patients with intrahepatic cholestasis of pregnancy (ICP).

	Healthy pregnant controls	ICP patients	P level
Serum total bile acids (µmol/L)	2.0±6.97	30.39±21.08	<1×10^−8^
***Primary bile acids and their conjugate forms (formed by synthesis in the liver)***			
CA	1.16±2.85	2.19±2.14	NS
CDCA	3.37±7.1	5.1±6.03	NS
GCA	0.31±0.83	2.83±5.86	NS
TCDCA	1.20±2.29	1.68±3.19	NS
GCDCA	0.0±0.0	2.36±4.2	0.03
TCA	0.44±1.17	3.45±5.56	NS
***Secondary bile acids and their conjugate forms (converted from primary by intestinal bacteria)***			
DCA	0.0±0.0	1.14±1.48	0.04
GDCA	2.13±3.96	2.72±6.31	NS
TDCA	4.52±4.33	4.51±4.27	NS
GLCA	0.0±0.0	3.09±6.57	NS
TLCA	0.0±0.0	1.51±2.23	0.01
***Tertiary bile acids (from Chenodeoxy cholic acid in liver)***			
UDCA	1.07±2.83	5.42±13.36	NS
TUDCA	11.69±8.25	8.36±6.44	NS

Results are expressed as mean ± SD, P-level indicates statistical significance using Mann-Whitney test.

CA (cholic acid), CDCA (chenodeoxycholic acid), DCA (deoxycholic acid), UDCA (**ursodeoxycholic acid**), GCA (glycocholic acid), GCDCA (glycochenodeoxycholic acid), GDCA (glycodeoxycholic acid), GLCA (glycolithocholic acid), GUCDA (glycoursoeodeoxycholic acid), TCA (taurocholic acid), TCDCA (taurochenodeoxycholic acid), TDCA (taurodeoxycholic acid), TLA (taurolithocholic acid), and TUDCA (tauroursodeoxycholic acid). Bile acids species are expressed as µM.

### Fetal Complications

No intrauterine fetal death was observed, but among ICP patients, fetal complications were registered in 35% of the neonates; these events were associated with higher total BA concentrations. Neonate Apgar score was significantly lower in babies whose mother had ICP, when compared with babies whose mother had a normal pregnancy, suggesting that the prevalence of transient cyanosis was higher in infant from ICP mothers; 3.5% of the full-term babies from mothers with ICP were small for gestational age.

### CpG Dinucleotides at the Gene Promoter of Nuclear Receptor Subfamily 1 Members and ATP-binding Cassette Transporter are Highly Methylated during Normal Gestation

We reasoned that the targeted measurement of promoter DNA methylation in late pregnancy might provide evidence about the environmental influences during the last trimester, and if they were correlated with the ICP phenotype, they would also provide an approach to demonstrate the role of maternal environment in predisposition to the disease.

**Figure 1:**
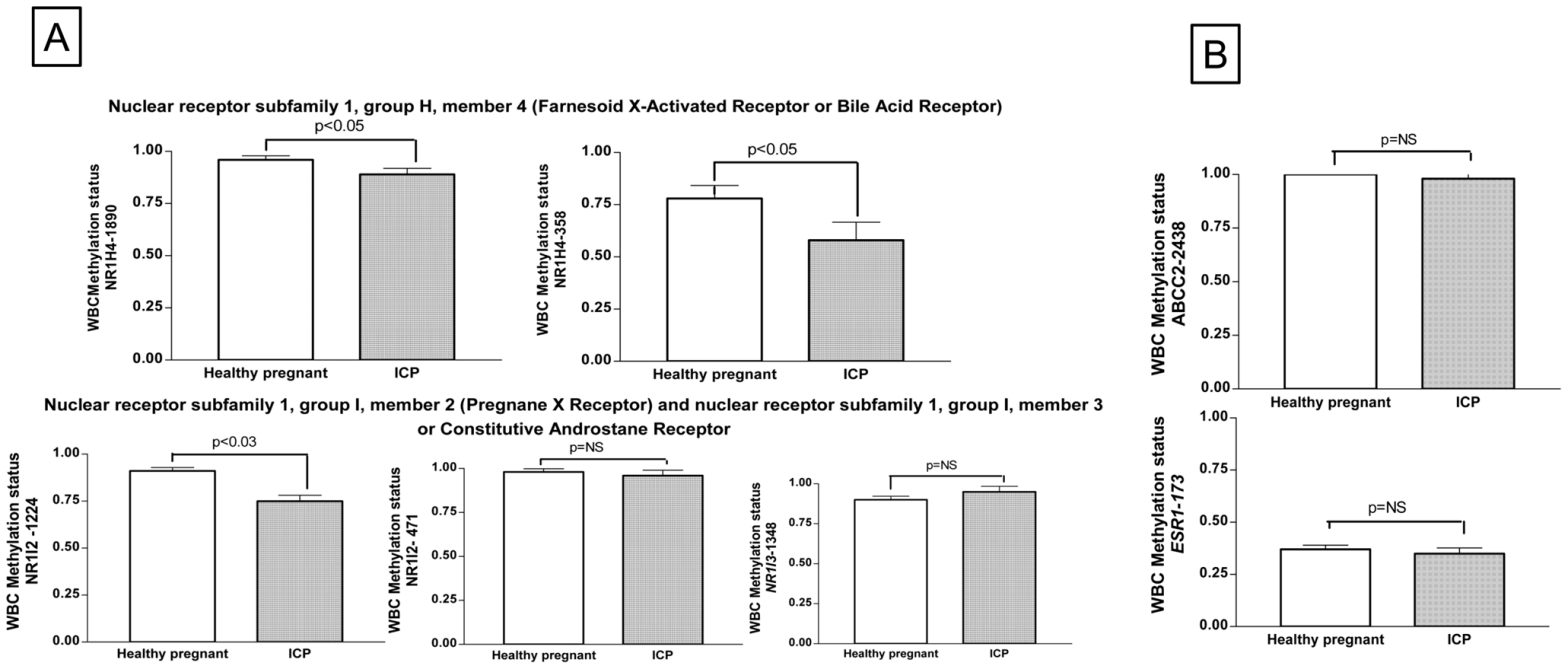
Exploration of methylation status at the 5′-cytosine residues of selected dinucleotide CpGs in the gene promoter of *FXR*/*NR1H4* (−358 and −1890), *PXR*/*NR1I2* (−471 and −1224), *NR1I3* (−2377), *ESR1* (−173), and *ABCC2* (−2438) located at positions relative to transcription starting site. A: Methylation levels of CpG dinucleotides at the gene promoter of nuclear receptor subfamily 1 members (*FXR*/*NR1H4*−358 and −1890, *PXR*/*NR1I2*−471 and −1224, and *NR1I3*−2377) in normal healthy pregnancy and in patients with ICP. B: Methylation levels of CpG dinucleotides at the gene promoter of ATP-binding cassette (ABC) transporter *ABCC2*-2438 and *ESR1*-173 in normal healthy pregnancy and in patients with ICP.

Of note, we observed that cytosine at the carbon-5 position in CpG dinucleotides of nuclear receptors *FXR*/*NR1H4*, *PXR*/*NR1I2,* and *NR1I3* was highly methylated during the third trimester of healthy pregnancy (>90%), except for the proximal site promoter at *FXR*/*NR1H4* that was almost 80% methylated (**Figure 1** **, A**). Indeed, we observed that the distal *FXR*/*NR1H4−*1890 promoter site was more methylated than the proximal promoter site (*NR1H4−358;* 96 vs. 78%, respectively; P<0.0000001), and a similar significant difference was observed for the two methylated sites for the *PXR*/*NR1I2* promoter (P<0.00002; **Figure 1** **, B**)**.** Likewise, promoter methylation of CpG dinucleotides of *ABCC2* was close to 100% in both cases and controls (**Figure 1** **, B**). On the contrary, CpG dinucleotides at the gene promoter of the nuclear hormone receptor *ESR1* were mostly unmethylated in both cases and controls, and the methylation status of the explored CpG site was close to 35% (**Figure 1** **, B**).

Interestingly, when we explored the status of gene promoter methylation according to the disease status, we observed significant differences for both the distal and proximal promoter CpG sites of the *FXR*/*NR1H4* and also *PXR* / *NR1I2*−1224 that were consistently less methylated in ICP cases, when compared with controls (**Figure 1** **, A**).

### Status of Promoter Methylation at Nuclear Receptors *FXR*/*NR1H4* and *PXR*/*NR1I2 (PXR)* is Highly Correlated with Bile Acid Profiling

We further examined the association between the status of methylation at CpG dinucleotides of the candidate genes and bile acid profiling. Of note, we observed that the methylation status of *FXR*/*NR1H4* at the distal −1890 CpG promoter site was significantly and positively correlated with serum levels of the primary bile acid chenodeoxycholic (CDCA) and the conjugated forms, taurochenodeoxycholic (TCDCA) and glycocholic acid (GCA), and also with the secondary bile acid taurolithocholic (TLCA) (**Table 3**). These findings suggest that the higher the methylation levels at the distal promoter CpG site, the higher the levels of conjugated BA, which are water soluble and are less prone to passive reabsorption once secreted into the small intestine. This seems to be a physiological mechanism in normal pregnancy.

**Table 3:** Statistical analysis of the correlation between methylation levels at the promoter of candidate genes and bile acid profiling.

Bile acid	Spearman rankcorrelation order R	P value
***FXR*** **/** ***NR1H4-1890***		
CDCA	0.57	0.003
TLCA	0.48	0.014
TCDCA	0.53	0.01
GCA	0.45	0.02
***FXR*** **/** ***NR1H4-358***		
CA	−0.40	0.03
DCA	−0.44	0.03
***PXR*** **/** ***NR1I2-1224***		
TLCA	0.42	0.032
GDCA	0.60	0.001
TDCA	0.44	0.008
***ABCC2-2438 (MRP)***		
TCA	0.52	0.01

Correlation between the status of promoter DNA methylation at CpG dinucleotides and bile acid profiling was tested using Spearman rank order correlation test.

Conversely, methylation levels at the proximal −358 CpG promoter site were significantly and negatively correlated with the primary cholic acid (CA) and the secondary deoxycholic acid (DCA) (**Table 3**), strongly suggesting that the lesser the methylation levels at the *FXR*/*NR1H4* proximal gene promoter, the higher the cholate synthesis by the liver and, thereby, the higher the level of the dehydroxylated from DCA converted by intestinal microbiota.

In addition, the status of *PXR*/*NR1I2* methylation at −1224 CpG site was also significantly and positively correlated with serum levels of secondary BAs, particularly, the glycol and taurine conjugated forms (TLCA, GDCA, and TDCA) (**Table 3**). Remarkably, methylation levels at *FXR*/*NR1H4*−1890 and *PXR*/*NR1I2*−1224 were significantly correlated with each other (R = 0.20, P = 0.001). Likewise, *PXR*/*NR1I2−*1224 and *PXR*/*NR1I2−*441 (R = 0.15, P = 0.02), *PXR*/*NR1I2−*441 and *ABCC2*−2438 (R = 0.25, P = 0.00008), and *PXR*/*NR1I2−*1224 and *NR1I3*−2377 (R = 0.16, P = 0.02) were also significantly correlated,

Regarding neonatal characteristics associated with the levels of DNA methylation at the gene promoters, an interesting finding was observed: *FXR*/*NR1H4* methylation at −1890 CpG site was significantly and negatively correlated with the anthropometrical variables of neonatal growth (cephalic perimeter R = −0.24, P = 0.02 and height R = −0.30, P = 0.01), suggesting that the lower the methylation levels, the higher the probability of children small for gestational age.

### Status of Methylation at the *FXR*/*NR1H4* and *PXR*/*NR1I2* during Gestation is Associated with Features of the Metabolic Syndrome

A recent report showed that ICP can program metabolic diseases in the offspring [17] suggesting that ICP is associated with an altered metabolic homeostasis that further affects the development of adult chronic diseases. In addition, recent evidence also show that *PXR*/*NR1I2* is implicated in metabolic syndrome (MetSynd) [18], [19].

To explore whether the maternal status of DNA methylation at gene promoters of nuclear receptors is associated with MetSynd-related phenotypes, we investigated the variables associated with insulin resistance (plasma insulin levels and HOMA-IR index) as well as those associated with blood pressure in the entire cohort of ICP and healthy pregnant women. Surprisingly, maternal status of methylation at *FXR*/*NR1H4*−1890 CpG site was significantly and inversely correlated with SABP (R = −0.32, P = 0.008), DABP (R = −0.30, P = 0.01) and fasting plasma insulin levels (R = −0.25, P = 0.01). The same pattern was observed with the methylation status of the *PXR*/*NR1I2*−1224 CpG site that was significantly and inversely correlated with both SABP (R = −0.28, P = 0.02) and DABP (R = −0.27, P = 0.03). Furthermore, the methylation status at the *FXR*/*NR1H4*-1890 CpG site significantly and inversely correlated with maternal body weight at term (R = −0.3, P = 0.006). Hence, partial demethylation of nuclear receptors might not only predispose to liver-related adverse pregnancy but also program in the offspring a systemic metabolic derangement that explains the findings observed in the Finland birth cohort [17].

### Epiallopregnanolone Sulfate, a Sulfated Progesterone Metabolite Decreases the *FXR*/*NR1H4* Transcriptional Activity but Marginally Affects the Promoter Methylation Levels at Selected CpG Sites

**Figure 2:**
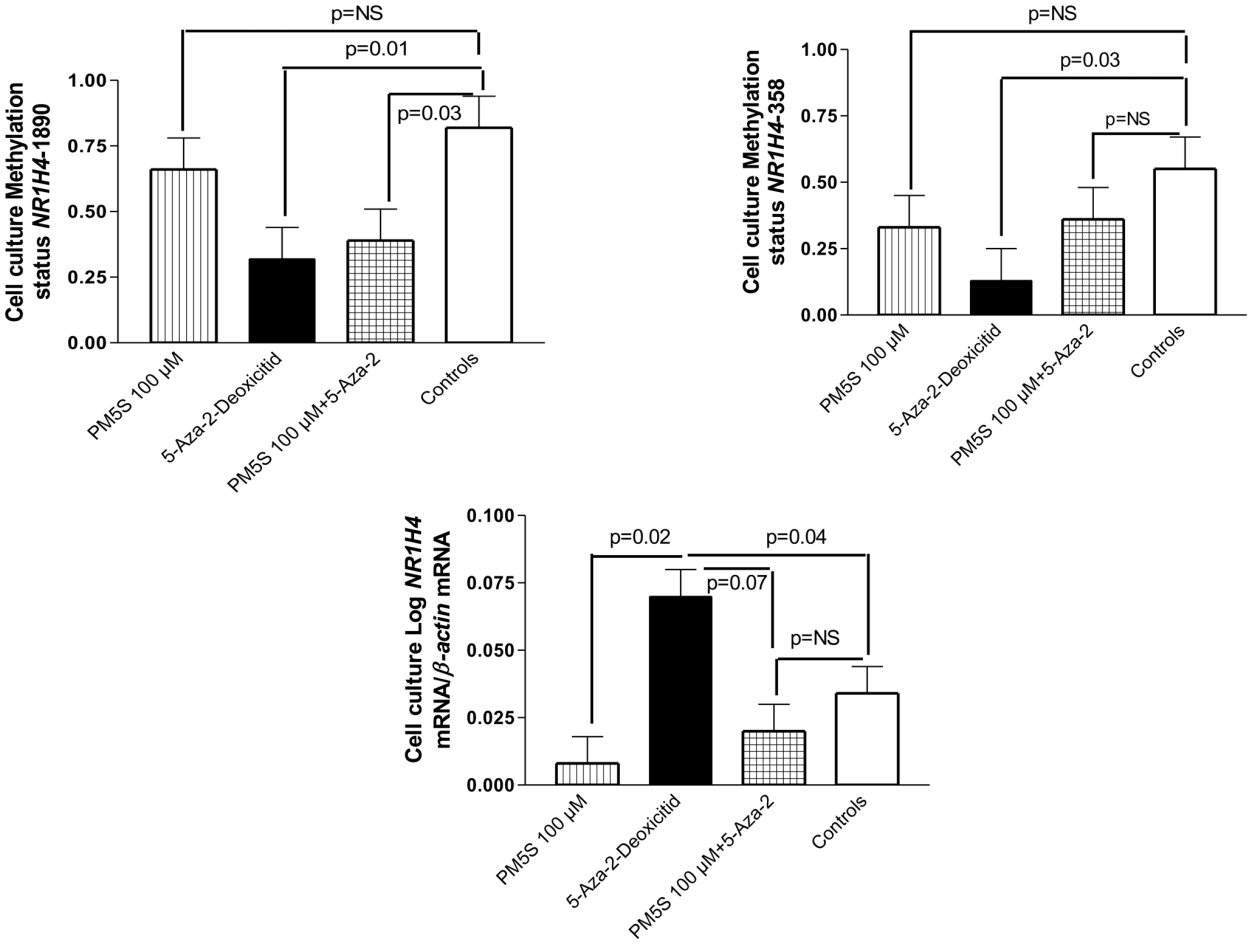
In vitro exploration of the effects of the progesterone metabolite, epiallopregnanolone sulfate PM5S, on the status of CpG methylation of the *FXR*/*NR1H4* gene promoter. Huh7 human hepatoma cells were treated with an agent that inhibits DNA methylation (5 µM 5-Aza-2′-deoxicitidine), a sulphated progesterone metabolite, a reported FXR inhibitor (PM5S, 5α-Pregnan-3β-OL-20-one sulfate, sodium salt, also known as epiallopregnanolone sulfate), and a combination of both agents. Controls cells did not receive any treatment. At the top of the figure, we show data about methylation changes at the proximal and distal *FXR*/*NR1H4* gene promoter, at the bottom we show changes in the *FXR*/*NR1H4* mRNA levels.

In light of a previous report that showed that epiallopregnanolone sulfate levels are above normal in patients with ICP and also competitively inhibit BA-mediated recruitment of co-factor motifs to the FXR-ligand-binding domain [20], we aimed to explore the effects of this progesterone metabolite on the methylation status of the two CpG sites at the *FXR*/*NR1H4* gene promoter. For this purpose, we used 5-Aza-2′-deoxycytidine (decitabine), an epigenetic modifier that inhibits DNA methyltransferase activity, which results in DNA demethylation and gene activation by remodeling “opening” chromatin. Of note, in control cells, we observed a highly methylated promoter at the distal *FXR*/*NR1H4*−1890 (∼80%) site, when compared with the proximal site (*FXR*/*NR1H4*−358) that was 50% methylated; these findings resemble those observed in the WBC of healthy pregnant women (**Figure 2**). As expected, exposure to decitabine was associated with a dramatic decrease in the methylation levels of both the promoter sites (**Figure 2**), and demethylation was associated with a significant increase in the transcriptional activity of *FXR*/*NR1H4* (**Figure 2**). These findings suggest that the selected CpG sites at the *NR1H4* promoter are highly sensitive to methylation modifications and are also associated with transcriptional regulation. Moreover, methylation levels at both the *FXR*/*NR1H4* promoter CpG sites were highly and positively correlated (R = 0.64, P = 0.0006).

When liver cells were exposed to epiallopregnanolone sulfate, we observed that despite being nonsignificant, there was a decrease in the level of promoter methylation at both the distal *FXR*/*NR1H4*−1890 (∼25%) and the proximal *FXR*/*NR1H4*−358 promoter sites (∼33%). Exposure to the progesterone metabolite was associated with a dramatic decrease in the *FXR*/*NR1H4* transcriptional gene activity (**Figure 2**).

## Discussion

In this study, we performed a targeted exploration of the status of DNA methylation at CpG promoter sites in candidate genes to explore whether the phenotype of ICP is modulated by epigenetic factors. We found evidence for changes from normal healthy pregnancy to cholestatic pregnancy that suggest that the disease phenotype is associated with a partial demethylation status that might modulate the development of the disease and BA profiling. Of note, a significant decrease in CpG methylation levels of 7.2% and 25% in the distal and proximal *FXR*/*NR1H4−*promoter sites, respectively, and 18.5% in the distal *PXR*/*NR1I2*−1224 site was observed in patients with ICP. Remarkably, we observed that the most contrasting changes accounted for the promoter methylation status of *FXR*/*NR1H4*; the distal and proximal *FXR*/*NR1H4* promoter sites exerted an opposite effect on the behavior of bile acid conjugation and cholate synthesis by the liver. Surprisingly, some effects were also observed regarding fetal outcomes, as methylation levels at *FXR*/*NR1H4* promoter sites significantly correlated with anthropometrical variables of neonatal growth.

Of note, methylation in the proximal and distal promoter might have differential effects because of the localization of different transcriptional regulatory elements along the promoter, which in the case of the proximal promoter can be the RNA polymerase binding site, TATA box, and transcription start site, and in the case of the distal one, can be enhancers, silencers, insulators, or locus control regions [21]. These two families of *cisacting* regulators can function either enhancing or repressing gene transcription [21]. Furthermore, methylation of distal regulatory sites is closely related to gene expression levels across the genome because they usually bind transcription factors in a methylation-dependent manner, and also carry particular chromatin marks of transcriptional enhancers as recently elegantly shown by Aran and coworkers [22]. Thus, the importance of proximal and distal promoter sites’ in the regulation of the transcriptional machinery may explain why even small differences in the methylation status of the explored CpG sites in our patients might have importance in physiological conditions.

To our knowledge, this is the first evidence in humans showing that ICP is associated with changes in the level of promoter DNA methylation in nuclear receptors involved in BA regulation and metabolism. Nevertheless, there are numerous and conclusive evidence about the role of both *FXR*/*NR1H4* and *PXR*/*NR1I2* in ICP. For instance, functional variants in the *FXR*/*NR1H4*gene were associated with ICP genetic susceptibility, and changes in the *FXR*/*NR1H4* placenta expression were observed in patients with ICP [23]. Furthermore, recent experimental studies in mice showed that inactivation of *fxr* by estrogen or its metabolites was associated with a procholestatic state [24], and epiallopregnanolone sulfate inhibits *fxr* action in vivo in mice and in vitro [20]. There is also evidence that *NR1I2 (PXR)* plays a role in ICP. In fact, it was shown that accumulation of bile acid precursors leads to *PXR*/*NR1I2* activation [25]. In addition, *PXR*/*NR1I2* regulates several genes involved in bile acid metabolism [26] and has been involved in the generic susceptibility of ICP. Our group reported that a gene variant in *PXR*/*NR1I2*was significantly associated with ICP and also with several disease-associated traits, suggesting a putative role of *PXR*/*NR1I2* variants in individual susceptibility to the disease, with the proportion of the total variation attributed to this variant being 7.8% [27].

Hence, to establish the relative contribution of hormonal factors to the *FXR*/*NR1H4* methylation status in patients with ICP, we performed an in vitro study that suggested that the progesterone metabolite epiallopregnanolone sulfate might not be involved in the observed changes at explored CpG sites in the *FXR* promoter. We observed a progesterone-associated inhibition of *FXR*/*NR1H4* gene transcription; the open chromatin state was associated with a dramatic increase in the mRNA levels, but when cells were exposed to progesterone in association with the demethylation agent, there was again a downregulation of the *FXR*/*NR1H4-*mRNA. Thus, we hypothesized that the disease phenotype might be associated with epigenetic changes other than those related to progesterone, which modulate the activity of the *FXR*/*NR1H4* loci.

A remarkable and unexpected finding of our study is an inverse association between the methylation levels at the *FXR*/*NR1H4* promoter and features of the MetSynd, including blood pressure, insulin resistance, and body weight gain during gestation. Indeed, these data are in agreement with recent evidence in humans indicating that cholestatic pregnancy can program metabolic diseases in the offspring because children of mothers with ICP were found to have features of MetSynd [17]. Furthermore, our data reinforce the concept of maternal programming of adult metabolic chronic diseases and emphasize the importance of effective and early intervention in ICP. Hence, methylation changes in a master gene that controls not only BA homeostasis [28] but also glucose and lipid metabolism [29] during adverse pregnancy may prime unexpected effects in the offspring of mothers suffering from ICP.

It is important to note that human liver samples cannot be collected in pregnant women, thus we cannot confirm that the status of DNA methylation at the *FXR*/*NR1H4* of blood genomic DNA behaves similarly than the liver. Nevertheless, there is evidence from human studies that *FXR*/*NR1H4* is expressed out of the digestive system [30], specifically previous studies showed that macrophages activation results in regulation of immune response and lipid metabolism by the activation of members of the nuclear receptor superfamily, including *FXR*/*NR1H4* [31], [32].

On the other hand, it is important to highlight that a previous study showed that during healthy pregnancy, the pattern of DNA methylation of the whole genome promoters significantly shifts from the first to the third trimester toward an overall progressive increase in the methylation levels at the end of gestation [33]. Indeed, Novakovic et al. noticed that CpG sites are susceptible to environmental changes associated with pregnancy, and minor methylation changes from 13% to 17% have biological impact and are critical for normal gestation [33]. Surprisingly, canonical pathways involved in arachidonic acid metabolism of xenobiotics by cytochrome P450 and circadian rhythm were in the higher methylation quartiles during the third trimester [33]. Consequently, our observation about a significant decrease (in the range of ∼7–25%) in the methylation levels of *FXR*/*NR1H4* and *PXR*/*NR1I2* might suggest at least an important impact on the control of gene expression of the nuclear receptor network and also putative changes in binding/ligand capability and interaction with other transcription factors, which is *per se* extremely complex [34].

A final comment should be added about the selection of the candidate genes in our study. We first focused our research on genes which encoded proteins critically regulate bile acid synthesis and transport in humans, such as *FXR*/*NR1H4* and *PXR*/*NR1I2.* In addition, *FXR*/*NR1H4* is a ligand-activated transcription factor by bile acids, such as chenodeoxycholic acid, lithocholic acid and deoxycholic acid [35]; also *FXR*/*NR1H4* activates the intestinal bile acid-binding protein (IBABP) and the transcription of bile acid salt export pump ABCB11 by directly recruiting histone methyltransferase CARM1 [36]. *FXR*/*NR1H4* also controls the expression of cytochrome P450 (CYP) 7A1; *PXR*/*NR1I2* and *NR1I3* are in the regulatory network of *FXR*/*NR1H4.* We decided to explore epigenetic changes in the promoter of *ABCC2* because in our population of patients, gene variants in *ABCC2*, including the rs3740066 located in exon 28, were significantly associated with the risk of ICP [10]. In an effort to explore an environmental factor that might explain the epigenetic modulation of the phenotype, we included in the analysis the estrogen receptor 1, which is a ligand-activated transcription factor with several domains for hormone binding. Constrains in the amount of the biological samples of the patients, limited us to further explore additional genes, for example, *MDR3/ABCB11* and *FIC1/ATP8B1*, which might be interesting to look at in future studies.

To conclude, some unresolved issues still remain to be explored; for example, some interindividual variation in DNA methylation may be explained by genetic polymorphisms among patients with ICP. Nonetheless, *FXR*/*NR1H4* is still a robust candidate gene to be explored in ICP [6] (**Figure S1)**.

## Supporting Information

Figure S1 Graphic illustration of gene/protein co-occurrence and their relatedness to biological concepts with the query “intrahepatic cholestasis of pregnancy.” Prediction was performed by PESCADOR (available at http://cbdm.mdc-berlin.de/tools/pescador/), a Web-based tool to assist large-scale integration text mining of biointeractions extracted from MEDLINE abstracts. The graph was constructed using the free available program MEDUSA, which is a Java application for visualizing and manipulating graphs of interaction (www.bork.embl.de/medusa). Our approach was used to explore by the use of a text-mining tool the available evidence about ICP in a systematic manner that further allows us to predict biomolecular interactions among relevant genes/proteins. The PESCADOR platform (Platform for Exploration of Significant Concepts AssociateD to co-Occurrences Relationships) allows selecting gene/protein co-occurrence pairs based on their relatedness to biological concepts bringing together, under a common perspective, protein interactions that have not been studied under the same research focus. After abstract tagging, 448 co-occurrences (gene/proteins) were retrieved, which were identified in 592 published abstracts. Interestingly, when these terms and interactions were displayed graphically, a hierarchical central hub appears centered on one gene/protein: *FXR*/*NR1H4.* This instrument shows that *FXR*/*NR1H4* is an excellent candidate gene to pursue in subsequent epigenetic studies on ICP. Also, these findings might help to understand the role of the *FXR*/*NR1H4* in the pathogenesis of the disease.
(TIF)

Table S1 Primer Sequences for DNA Methylation promoter detection and messenger gene abundance measurement by real-time PCR.
(DOC)

## References

[1] Rioseco AJ, Ivankovic MB, Manzur A, Hamed F, Kato SR, et al (1994) Intrahepatic cholestasis of pregnancy: a retrospective case-control study of perinatal outcome. Am J Obstet Gynecol 170: 890–895. 8141222 10.1016/s0002-9378(94)70304-3

[2] Laatikainen T, Tulenheimo A (1984) Maternal serum bile acid levels and fetal distress in cholestasis of pregnancy. Int J Gynaecol Obstet 22: 91–94. 6145644 10.1016/0020-7292(84)90019-5

[3] Castano G, Lucangioli S, Sookoian S, Mesquida M, Lemberg A, et al (2006) Bile acid profiles by capillary electrophoresis in intrahepatic cholestasis of pregnancy. Clin Sci (Lond) 110: 459–465. 16356162 10.1042/CS20050302

[4] Geenes V, Williamson C (2009) Intrahepatic cholestasis of pregnancy. World J Gastroenterol 15: 2049–2066. 19418576 10.3748/wjg.15.2049 PMC2678574

[5] Pauli-Magnus C, Meier PJ, Stieger B (2010) Genetic determinants of drug-induced cholestasis and intrahepatic cholestasis of pregnancy. Semin Liver Dis 30: 147–159. 20422497 10.1055/s-0030-1253224

[6] Sookoian S, Pirola CJ (2012) Genetic determinants of acquired cholestasis: a systems biology approach. Front Biosci 17 206–220: 3922. 10.2741/3922 22201739

[7] Reyes H, Gonzalez MC, Ribalta J, Aburto H, Matus C, et al (1978) Prevalence of intrahepatic cholestasis of pregnancy in Chile. Ann Intern Med 88: 487–493. 637428 10.7326/0003-4819-88-4-487

[8] Reyes H, Baez ME, Gonzalez MC, Hernandez I, Palma J, et al (2000) Selenium, zinc and copper plasma levels in intrahepatic cholestasis of pregnancy, in normal pregnancies and in healthy individuals, in Chile. J Hepatol 32 542–549: S0168–8278(00)80214-7. 10.1016/s0168-8278(00)80214-7 10782901

[9] Reyes H, Zapata R, Hernandez I, Gotteland M, Sandoval L, et al (2006) Is a leaky gut involved in the pathogenesis of intrahepatic cholestasis of pregnancy? Hepatology 43: 715–722. 16557543 10.1002/hep.21099

[10] Sookoian S, Castano G, Burgueno A, Gianotti TF, Pirola CJ (2008) Association of the multidrug-resistance-associated protein gene (ABCC2) variants with intrahepatic cholestasis of pregnancy. J Hepatol 48 125–132: S0168–8278(07)00563-6. 10.1016/j.jhep.2007.08.015 17997497

[11] Apgar V (1966) The newborn (Apgar) scoring system. Reflections and advice. Pediatr Clin North Am 13: 645–650. 5946299 10.1016/s0031-3955(16)31874-0

[12] Capurro H, Konichezky S, Fonseca D, Caldeyro-Barcia R (1978) A simplified method for diagnosis of gestational age in the newborn infant. J Pediatr 93: 120–122. 650322 10.1016/s0022-3476(78)80621-0

[13] San PM, Grandi C, Larguia M, Solana C (2001) [Standard of birth weight for gestational age in 55706 healthy newborns in a public maternity of Buenos Aires]. Medicina (B Aires) 61: 15–22. 11265618

[14] Kawasaki ES (1990) Sample preparation from blood, cells, and other fluids. In: Innis MA, Gelfand DH, Sninsky JJ, White TJ, editors. PCR Protocols. A guide to Methods and Applications. San diego: Academic Press, INC. 146–152.

[15] Singer-Sam J, Grant M, LeBon JM, Okuyama K, Chapman V, et al (1990) Use of a HpaII-polymerase chain reaction assay to study DNA methylation in the Pgk-1 CpG island of mouse embryos at the time of X-chromosome inactivation. Mol Cell Biol 10: 4987–4989. 1697035 10.1128/mcb.10.9.4987-4989.1990 PMC361130

[16] Singer-Sam J, LeBon JM, Tanguay RL, Riggs AD (1990) A quantitative HpaII-PCR assay to measure methylation of DNA from a small number of cells. Nucleic Acids Res 18: 687. 1689825 10.1093/nar/18.3.687 PMC333509

[17] Papacleovoulou G, Abu-Hayyeh S, Nikolopoulou E, Briz O, Owen B, et al (2013) Maternal cholestasis during pregnancy programs metabolic disease in offspring. The Journal of Clinical Investigation 123: 3172–3181. 23934127 10.1172/JCI68927 PMC3696570

[18] He J, Gao J, Xu M, Ren S, Stefanovic-Racic M, et al (2013) PXR ablation alleviates diet-induced and genetic obesity and insulin resistance in mice. Diabetes 62: 1876–1887. 23349477 10.2337/db12-1039 PMC3661619

[19] Rysa J, Buler M, Savolainen MJ, Ruskoaho H, Hakkola J, et al (2013) Pregnane X receptor agonists impair postprandial glucose tolerance. Clin Pharmacol Ther 93: 556–563. 23588309 10.1038/clpt.2013.48

[20] Abu-Hayyeh S, Papacleovoulou G, Lovgren-Sandblom A, Tahir M, Oduwole O, et al (2013) Intrahepatic cholestasis of pregnancy levels of sulfated progesterone metabolites inhibit FXR resulting in a pro-cholestatic phenotype. Hepatology 57: 716–26. 22961653 10.1002/hep.26055 PMC3592994

[21] Maston GA, Evans SK, Green MR (2006) Transcriptional regulatory elements in the human genome. Annu Rev Genomics Hum Genet 7: 29–59. 16719718 10.1146/annurev.genom.7.080505.115623

[22] Aran D, Sabato S, Hellman A (2013) DNA methylation of distal regulatory sites characterizes dysregulation of cancer genes. Genome Biol 14 R21: gb–2013-14-3-r21. 10.1186/gb-2013-14-3-r21 PMC4053839 23497655

[23] Geenes VL, Dixon PH, Chambers J, Raguz S, Marin JJ, et al (2011) Characterisation of the nuclear receptors FXR, PXR and CAR in normal and cholestatic placenta. Placenta 32: 535–537. 21596433 10.1016/j.placenta.2011.04.014

[24] Milona A, Owen BM, Cobbold JF, Willemsen EC, Cox IJ, et al (2010) Raised hepatic bile acid concentrations during pregnancy in mice are associated with reduced farnesoid X receptor function. Hepatology 52: 1341–1349. 20842631 10.1002/hep.23849

[25] Goodwin B, Gauthier KC, Umetani M, Watson MA, Lochansky MI, et al (2003) Identification of bile acid precursors as endogenous ligands for the nuclear xenobiotic pregnane X receptor. Proc Natl Acad Sci U S A 100: 223–228. 12509506 10.1073/pnas.0237082100 PMC140933

[26] Kliewer SA, Goodwin B, Willson TM (2002) The nuclear pregnane X receptor: a key regulator of xenobiotic metabolism. Endocr Rev 23: 687–702. 12372848 10.1210/er.2001-0038

[27] Castano G, Burgueno A, Fernandez GT, Pirola CJ, Sookoian S (2010) The influence of common gene variants of the xenobiotic receptor (PXR) in genetic susceptibility to intrahepatic cholestasis of pregnancy. Aliment Pharmacol Ther 31: 583–592. 19958310 10.1111/j.1365-2036.2009.04210.x

[28] Halilbasic E, Claudel T, Trauner M (2013) Bile acid transporters and regulatory nuclear receptors in the liver and beyond. J Hepatol 58: 155–168. 22885388 10.1016/j.jhep.2012.08.002 PMC3526785

[29] Claudel T, Staels B, Kuipers F (2005) The Farnesoid X receptor: a molecular link between bile acid and lipid and glucose metabolism. Arterioscler Thromb Vasc Biol 25: 2020–2030. 16037564 10.1161/01.ATV.0000178994.21828.a7

[30] Bishop-Bailey D (2004) FXR as a novel therapeutic target for vascular disease. Drug News Perspect 17 499–504: 506. 10.1358/dnp.2004.17.8.863693 15605109

[31] Glass CK, Saijo K (2010) Nuclear receptor transrepression pathways that regulate inflammation in macrophages and T cells. Nat Rev Immunol 10: 365–376. 20414208 10.1038/nri2748

[32] Renga B, Francisci D, D’Amore C, Schiaroli E, Mencarelli A, et al (2012) The HIV matrix protein p17 subverts nuclear receptors expression and induces a STAT1-dependent proinflammatory phenotype in monocytes. PLoS One 7: e35924 10.1371/journal.pone.0035924 [doi];PONE-D-11-25560 [pii] 22558273 10.1371/journal.pone.0035924 PMC3340403

[33] Novakovic B, Yuen RK, Gordon L, Penaherrera MS, Sharkey A, et al (2011) Evidence for widespread changes in promoter methylation profile in human placenta in response to increasing gestational age and environmental/stochastic factors. BMC Genomics 12 529: 1471–2164-12-529. 10.1186/1471-2164-12-529 PMC3216976 22032438

[34] Lee J, Seok S, Yu P, Kim K, Smith Z, et al (2012) Genomic analysis of hepatic farnesoid X receptor binding sites reveals altered binding in obesity and direct gene repression by farnesoid X receptor in mice. Hepatology 56: 108–117. 22278336 10.1002/hep.25609 PMC3343176

[35] Downes M, Verdecia MA, Roecker AJ, Hughes R, Hogenesch JB, et al (2003) A chemical, genetic, and structural analysis of the nuclear bile acid receptor FXR. Mol Cell 11: 1079–1092. 12718892 10.1016/s1097-2765(03)00104-7 PMC6179153

[36] Ananthanarayanan M, Li S, Balasubramaniyan N, Suchy FJ, Walsh MJ (2004) Ligand-dependent activation of the farnesoid X-receptor directs arginine methylation of histone H3 by CARM1. J Biol Chem 279: 54348–54357. 15471871 10.1074/jbc.M410021200

