# Supplementary figures and images for: Promoter DNA Methylation of Farnesoid X Receptor and Pregnane X Receptor Modulates the Intrahepatic Cholestasis of Pregnancy Phenotype
Source: PLoS One. 2014 Jan 31;9(1):e87697. doi: 10.1371/journal.pone.0087697 (PMC3909199; doi:10.1371/journal.pone.0087697)

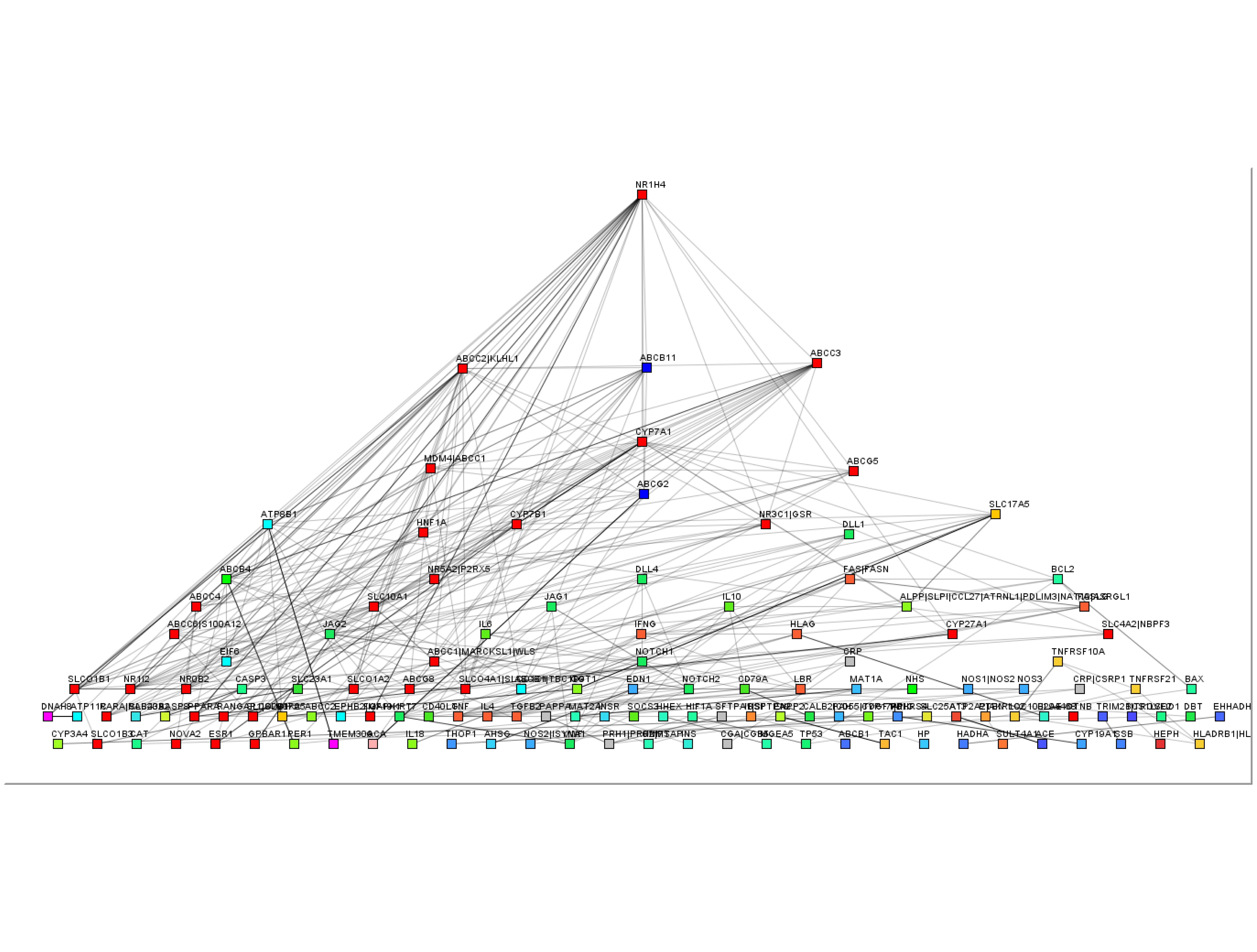

Supplement: Figure S1 — Graphic illustration of gene/protein co-occurrence and their relatedness to biological concepts with the query “intrahepatic cholestasis of pregnancy.” Prediction was performed by PESCADOR (available at http://cbdm.mdc-berlin.de/tools/pescador/), a Web-based tool to assist large-scale integration text mining of biointeractions extracted from MEDLINE abstracts. The graph was constructed using the free available program MEDUSA, which is a Java application for visualizing and manipulating graphs of interaction (www.bork.embl.de/medusa). Our approach was used to explore by the use of a text-mining tool the available evidence about ICP in a systematic manner that further allows us to predict biomolecular interactions among relevant genes/proteins. The PESCADOR platform (Platform for Exploration of Significant Concepts AssociateD to co-Occurrences Relationships) allows selecting gene/protein co-occurrence pairs based on their relatedness to biological concepts bringing together, under a common perspective, protein interactions that have not been studied under the same research focus. After abstract tagging, 448 co-occurrences (gene/proteins) were retrieved, which were identified in 592 published abstracts. Interestingly, when these terms and interactions were displayed graphically, a hierarchical central hub appears centered on one gene/protein: FXR/NR1H4. This instrument shows that FXR/NR1H4 is an excellent candidate gene to pursue in subsequent epigenetic studies on ICP. Also, these findings might help to understand the role of the FXR/NR1H4 in the pathogenesis of the disease. (TIF) [file pone.0087697.s001.tif]
