# Supplementary material for: Promoter DNA Methylation of Farnesoid X Receptor and Pregnane X Receptor Modulates the Intrahepatic Cholestasis of Pregnancy Phenotype
Source: PLoS One. 2014 Jan 31;9(1):e87697. doi: 10.1371/journal.pone.0087697 (PMC3909199; doi:10.1371/journal.pone.0087697)
Supplement: Table S1 — Primer Sequences for DNA Methylation promoter detection and messenger gene abundance measurement by real-time PCR. (DOC) [file pone.0087697.s002.doc]

**Table S1**

**Primer Sequences for DNA Methylation promoter detection and messenger gene abundance measurement by real-time PCR**

|  | **The primers sequences for methylation-specific polymerase chain reaction** | |
| --- | --- | --- |
| **Gene** | **Forward primer 5’→3’** | **Reverse primer 5’→3’** |
| *NR1H4-358* | GGCTCCCAGCTTCTAGTTCA | TGGGCACCCGTATTTCTGTA |
| *NR1H4-1890* | CAGATGGGGAAACTGAGACAG | TCCAGCAGAGATGGAGACAA |
| *NR1I2-471* | CGGCTCCTTGGTAAAGCTAC | AACCATACCTGCTTGGTGGT |
| *NR1I2-1224* | GGACTCTTCAGCTCCCCTCT | TGACCCCACCACTTTGATCT |
| *ESR1-173* | GGCACATAAGGCAGCACATT | GCACGAGGATCTGCTAAAGG |
| *ABCC2-2438* | TCTCGATCTCCTGACCTCGT | TCACAATTTACTGGCGCAGA |
| *NR1I3-2377* | ccagccaattctttgcattt | tgttggatttgagggggtag |
|  | **The primer sequences for mRNA gene expression** | |
| **Gene** | **Forward primer 5’→3’** | **Reverse primer 5’→3’** |
| *NR1H4* | AGAGATGGGAATGTTGGCTGA | TCACTTGTCGCAAGTCACGA |
